# Bend-Direction and Rotation Plastic Optical Fiber Sensor

**DOI:** 10.3390/s20185405

**Published:** 2020-09-21

**Authors:** Demetrio Sartiano, Thomas Geernaert, Elena Torres Roca, Salvador Sales

**Affiliations:** 1 Institute of Telecommunications and Multimedia Applications (iTEAM), Universitat Politècnica de València, Camino de Vera, s/n 46022 Valencia, Spain; ssales@dcom.upv.es; 2 Brussels Photonics (B-PHOT), Department of Applied Physics and Photonics, Vrije Universiteit Brussel and Flanders Make, Pleinlaan 2, B-1050 Brussels, Belgium; thomas.geernaert@vub.be; 3 Textile Research Institute (AITEX), Plaza Emilio Sala, 1, 03801 Alcoy, Spain; etorres@aitex.es

**Keywords:** plastic optical fiber, optical fiber sensor, extruded optical fiber, bending sensor

## Abstract

A plastic filament of poly (methyl methacrylate) (PMMA) was fabricated by extrusion. The mode confinement was simulated using numerical software. The idea is to study how the light intensity changes inside the plastic optical fiber (POF) when a bending in multiple directions is applied. The results obtained from the simulation were compared to the experimental observations. The non-circular shape of the POF allows sensing a rotation applied as well. The angle of rotation was obtained processing two images of the end facet of the fiber (one with the fiber in a reference position and one with the rotated fiber), using an intensity-based automatic image registration. The accuracy in the rotation calculation was of 0.01°.

## 1. Introduction

Fiber-based sensors have played an important role in many recent applications, including bridge security monitoring [1], biochemical sensing [2], gas detectors [3], and power system monitoring [4], due to their advantages, such as small size, immunity to electromagnetic interference, remote sensing capability, and high sensitivity. Novel fiber sensors are implemented by innovating the fiber grating structure or inscribing gratings in specialty fibers instead of standard single-mode fibers (SMF). These designs are focused on solving the problem of cross-sensitivity between temperature and strain and implementing multi-parameter fiber sensors for new measurands. Baiou Guan et al. [5] used a superstructure fiber Bragg grating (FBG) to simultaneously measure strain and temperature; Maoqing Chen et al. [6] combined a micro extrinsic Fabry–Perot interferometer with an etched FBG for the simultaneous measurement of strain and magnetic field. Multicore fibers (MCF) have also been used in the sensing field: David Barrera et al. [7] inscribed a tilted fiber Bragg grating (TFBG) in a seven-core fiber by Argon-ion laser to measure strain, curvature direction and magnitude, and external refractive index (RI). Strongly coupled MCF is an alternative to grating-based sensors: [8] reported a strain sensor implemented with an MCF with cores closer together than in a common MCF. The interference between two supermodes excited in the MCF has been used to obtain an interferometric strain sensor. Multi-parameter sensors for rotation and bending are reported in the literature as well [9,10,11,12,13,14,15].

Plastic optical fibers offer a valid cheap alternative to glass fibers in certain applications, since they are manufactured with low-cost materials and processes and have good mechanical properties. There are various techniques presented in the literature to use plastic optical fiber (POF) as sensors. After the inscription of the first FBG in a step index POF in 1999 [16], these fiber-based sensors were adopted for strain and temperature, demonstrating a sensitivity of 1.48 pm/με and 55 pm/°C [17,18]. The inscription of grating in POF with a resonant wavelength in the C-band allows using a commercial FBG interrogator, coupling a glass fiber connector with the POF using UV resin. In recent works, pulse wave and blood pressure monitoring were demonstrated using this method [19]. Other techniques used for glass fiber sensing that have been adopted for POFs are the optical reflectometries in frequency and time domain. The sensing characteristics of POF with optical time domain reflectometry (OTDR) were investigated, as mechanical splice, small bend torsional strain, axial strain, and temperature in [20]. Since then, POF and OTDR were adopted for a wide range of applications, as pH sensing [21] or long-distance intrusion sensing over 60 km of fiber [22]. All the previous applications required the use of bulky instrumentation to interrogate the sensors, and it should be taken in account also that the inscription of gratings in POF required the use of ultra-fast laser [23,24,25]. Plastic fibers are preferable in applications in which high levels of mechanical strain are applied to the sensor, since the Young’s modulus value of bulk poly(methyl methacrylate) is 3.2 GPa (while it is 72 GPa in silica fibers) and it has high elastic deformation limits (10%). This polymer is also impact and vibration-resistant and has a lower density (1.195 kg/m^−3^) than silica [26]. The material most frequently used to make POFs is poly (methyl methacrylate) (PMMA) thermoplastic polymer, which is commonly known as Plexiglas^®^; it has a typical refractive index of 1.492 (Plexiglas^®^ 6N at 589 nm at 23 °C) [27], and it is generally manufactured in two steps. First, a pre-formed solid cylindrical rod is made approximately 0.5–1 m long and several centimeters in diameter. The structure of this preform determines the core and cladding refractive index profiles. The second step consists of extruding the preform, which yields a POF between half a kilometer and several kilometers long [28]. Recent advances in polymer technology have allowed fabricating plastic optical fiber for sensing. The advantages of optical metrology with plastic optical fiber have attracted the attention of the scientific community, as they provide lower-cost systems than conventional technologies [27]. POF-based curvature sensors were developed mostly as intensity sensors: for example, in [29,30], the core of the fiber is exposed, abrading part of the cladding to enhance the irradiated light when a bend is applied. These sensors were used to monitor human joint and spine curvature [31,32], as curvature sensors for structure health monitoring [33], or as a sensor to monitor breathing [34]; nano displacement was sensed as well using a double core fiber and an interferometric approach [35].

In this work, a non-circular optical fiber was fabricated, consisting of a three-lobe PMMA fiber core without cladding. The filament was directly extruded from plastic pellets, and the three-lobe shape was obtained using a custom-shaped extrusion die. The three-lobe shape was conceived to achieve a low-cost optical fiber bend direction and rotation sensor. The bend direction sensing principle is made observing the change in the light field distribution inside the three-lobe core when the plastic filament is bent. The circular asymmetry of the fiber allows retrieving the direction of bending and the rotation angle. The sensor is interrogated in transmission using a red LED (645 nm) and a charge coupled device (CCD) placed in front of the end facet of the fiber. 

The paper is structured as follows: the first section contains a brief presentation of the fabrication process of the POF, together with results of guided mode simulations in the obtained structure. The second section provides the experimental results obtained from the fiber, while the conclusions are given in the last section.

## 2. Fabrication of Three-Lobe Plastic Optical Fiber

A transparent material allows light to be transmitted through the material matrix with minimum attenuation. In semi-crystalline polymers, such as PMMA, transparency is directly related to the crystallinity of the polymer [36]. Crystallinity is an intrinsic property of the polymeric material where molecular chains are aligned and folded together to form ordered regions called lamellae, which compose larger spheroidal structures named spherulites [37], and the refractive index of crystalline regions is higher than that of amorphous regions. When a light ray pass through an ordered region or spherulites with larger size than the wavelength of visible light (0.4–0.7 μm), a light scattering is produced. Therefore, amorphous materials will transmit the light in a higher level compared with crystalline or semi-crystalline materials and a decrease in crystallinity in a semi-crystalline polymer enhances the clarity. The processability of the polymer can modify the polymer crystallinity; however, a reduction in crystallinity can result in a decrease in strength, stiffness, and resistance to bend.

**Figure 1:**
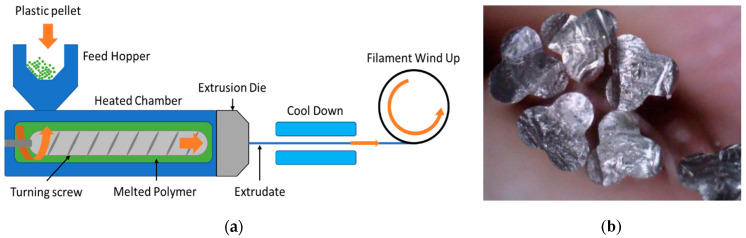
(**a**) Scheme of the extrusion system: screw velocity and velocity of the wind-up machine were optimized to find the best performance in terms of optical losses. In this case, the filaments were air-cooled, since poly (methyl methacrylate) (PMMA) is water absorbent. (**b**) Photo of the cross-sections of the obtained plastic optical fibers.

A scheme of the extrusion system used to fabricate the POF is shown in Figure 1a. The process begins by melting the polymer using a standard extruder. Generally, barrier flight screws with a mixer on the end are used to drive the polymer pellet to the screw. The melting process is mostly accomplished by shear heating from the turning of the screw and by different temperature-controlled zones placed along the length of the extruder. The extrusion process is controlled by a pump that controls the speed of the extruder screw to give a constant polymer flow rate. At the end of the spin line, the spinneret or die is found, which contains one or more holes of a specific geometry and diameter in order to provide the polymer with the desired shape. Since the filament will be drawn in a further process, the final diameter will be different than the diameter at this point. Once the filament has been extruded, the polymer needs to be cooled down in a water bath or air chamber. After the polymer filament is cooled down, it is collected by a rotating spool. Both the material’s pressure and cooling temperature influence the PMMA crystallinity, which directly impacts the attenuation of the light transmitted into the POF. The machinery normally used to draw fiber for textile was employed to fabricate POF using a custom extrusion die to obtain a three-lobe shape (Figure 1b). The fabrication parameters were optimized to minimize the POF’s optical losses.

Three fiber samples were fabricated with two replicates for each sample. The parameters were different grades of PMMA (whose main properties are reported in Table 1), modification of the flow rate by varying pump velocity (rpm), and the draw ratio controlled by the spool speed (m/min) to obtain samples of constant thickness. The samples were air-dried at room temperature.

Table 2 shows the fiber losses measured using the cut-back method [38]. We took one meter of fiber and we cut off 10 cm for each step of the cut-back measurement. The light source used is an HeNe (Thorlabs, Netwon, N J, USA) laser emitting at 632.8 nm. We considered two different plastic fiber samples, since we expect to have a certain variability between the fiber spools due to the fabrication process. The lowest optical loss was obtained with high draw ratios at high spool speed. The higher variation for the losses measured for Sample 2 is due to the lower PMMA pump velocity at a higher spool speed that facilitates the onset of inhomogeneity in the fiber spool.

## 3. Sensor Design and Simulations

**Figure 2:**
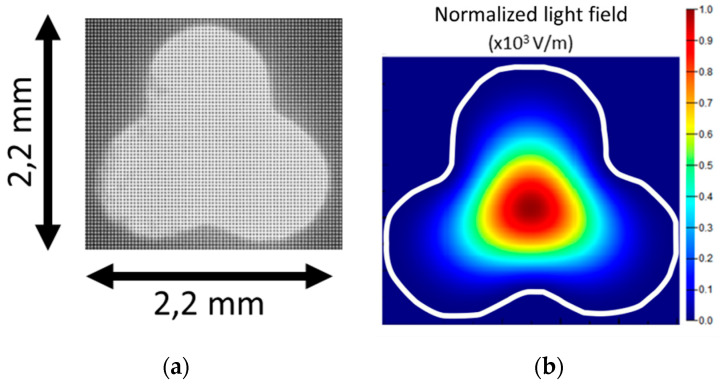
(**a**) Optical microscope image of the cross-section of the three-lobe plastic optical fiber. (**b**) Simulated light field intensity of the fundamental mode at 645 nm.

The transversal section of the three-lobe fiber was captured with an optical microscope (The Imaging Source Europe GmbH, Bremen, Germany) (Figure 2a), processed, and then imported into Lumerical MODE software. Mode simulations were performed to calculate the light field distribution of the lowest order modes in both the straight and bent fiber. Figure 2b shows the light field intensity of the lowest order fiber mode simulated in the straight fiber.

**Figure 3:**
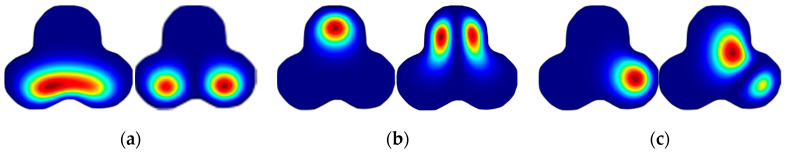
Light field intensity at 645 nm of the two lowest order modes calculated in the imported three-lobe structure cross-section for bending with curvature center placed: (**a**) on the upper surface of the fiber; (**b**) on the lower surface of the fiber; (**c**) on the right-hand surface of the fiber.

As observed in previous calculations and simulations on bent fibers, bending tends to distort the fiber modes and causes them to shift away from the center of curvature [39]. In case of a bent waveguide, Lumerical MODE software will solve for modes of the form:

(1) $$E\left(\rho ,\theta ,z\right)=E_{j}\left(\rho ,y\right)e^{i\beta_{j}\theta }$$

where *β_j_* depicts the angular propagation constant of the *j*_th_ mode and has units of inverse radian, and (*ρ*, *θ*, *y*) represents a cylindrical coordinate system. This constant is proportional to the effective index of the mode and the bending radius [40]. The simulated light field intensity for the lowest order modes under three-directional bending with a radius of 10 m is shown in Figure 3; the scale is the same as in Figure 2a. The simulations confirmed that the guided modes in the bent three-lobe fiber shifted away from the center of curvature.

The intensity variation that shifts away from the bend center can be used to estimate the bending direction. The calculation of the weighted centroid is the algorithm chosen to process the intensity image and give an estimation of the curvature direction; the centroid is calculated using the intensity of the pixel as weight. For a 2D image N×M, it is possible to calculate the centroid coordinates using the formula (with *I*: intensity and *x*/*y*: horizontal/vertical coordinates):

(2) $$x_{f}=\frac{\sum_{j=1}^{M}\sum_{i=1}^{N}I_{i,j}x_{i}}{\sum_{j=1}^{M}\sum_{i=1}^{N}I_{i,j}};\;y_{f}=\frac{\sum_{i=1}^{N}\sum_{j=1}^{M}I_{i,j}y_{j}}{\sum_{j=1}^{M}\sum_{i=1}^{N}I_{i,j}}$$

## 4. Experimental Results

**Figure 4:**
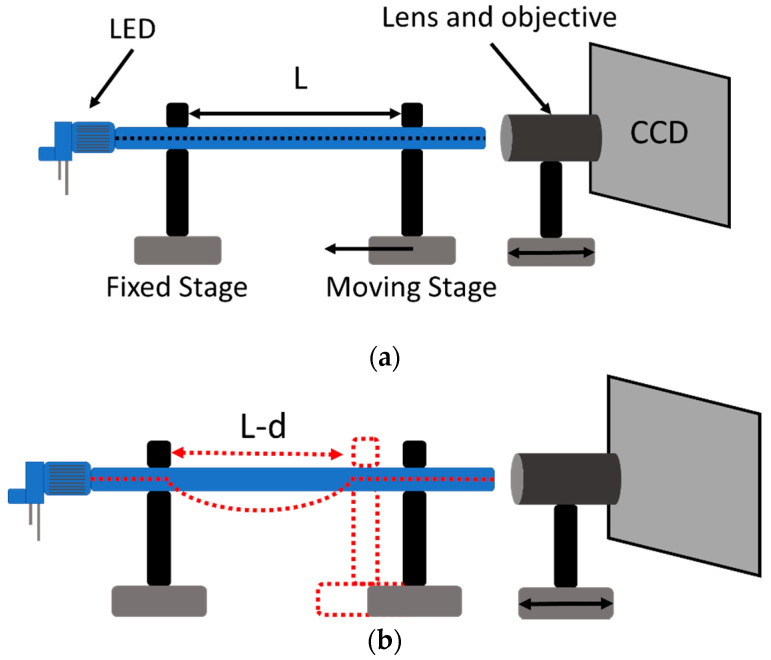
Schematic representation of the experimental setup (**a**) and method used to obtain constant curvature (**b**).

The setup used to interrogate the three-lobe fiber in transmission was implemented as follows: the POF was fixed to a translational stage to allow controlled bending whilst observing the light intensity distribution inside the core. Sensors based on intensity variation were among the first detection schemes used in optical fiber sensors and can be considered the simplest method in terms of operating principle and instrumentation. In general, experimental setups include a light source, the optical fiber, and a photodetector or an optical spectrum analyzer. Several solutions for miniaturized solid-state sources and photodetectors are now commercially available, allowing the design and development of robust and portable acquisition systems. This is a suitable solution for engineering applications in which the accuracy of the power signal measurement is not critical. The advantages of this method are its ease of implementation, good price/quality ratio, and simple signal processing [41]. The plastic fiber was interrogated in transmission using an LED emitting at 645 nm (IF-E96 from Industrial Fiberoptics, Tempe, AZ, USA) and a CCD camera (The Imaging Source Europe GmbH, Bremen, Germany) (Figure 4a).

In this case, we need to use a two-dimensional light sensor, because the light intensity distribution has to be monitored—and not only the intensity, as is usual for intensity-based sensors. A sample of 20 cm was fixed between the two holders and a curvature with a ray of approximately 1 m was applied (Figure 4b). The constant curvature was obtained using a moving stage, and the curvature was approximated using the formula [42]:

(3) $$sin\left(\frac{LC}{2}\right)=\frac{\left(L-D\right)C}{2}.$$

**Figure 5:**
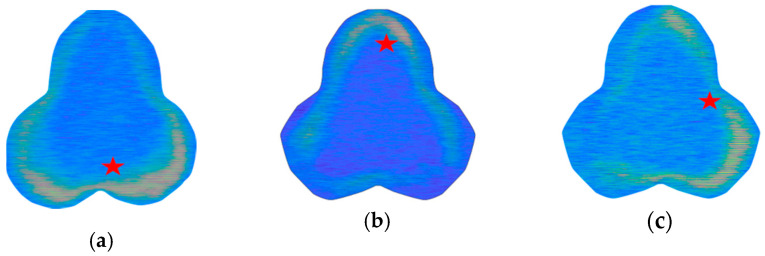
Images collected with the charge coupled device (CCD) camera after bending the three-lobe plastic fiber. Red stars indicate the position of the weighted centroid found from the converted gray-scale image. The curvature center placed: (**a**) on the upper surface of the fiber; (**b**) on the lower surface of the fiber; (**c**) on the right-hand surface of the fiber.

The images were captured before and after bending and then compared on Matlab software. The resultant images were converted to gray scale, and the weigthed centroids were found. Figure 5 shows three images obtained for bending orientation along the x and y axes (red stars represent the weighted centroid). A light intensity variation is clearly observable in the exterior of the fiber, which is caused by the shift of the light field intensity due to the bending applied. The effects of bending on lower order modes was confirmed observing the light intensity distribution in the experimental case. It was not possible to observe any variation with the curvature radius, while an analysis of the effect of bending on higher order modes could provide a quantitative information about the radius of curvature, but this analysis is out of the scope of this work.

**Figure 6:**
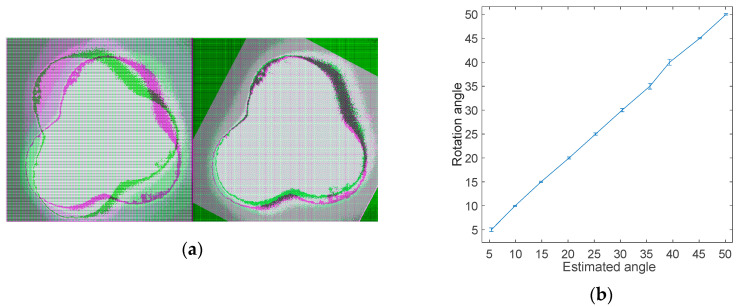
(**a**) Reference and rotated image superimposed before and after the registration algorithm is applied. (**b**) Comparison between the retrieved angles using the registration algorithm and the rotation angles applied. The error bars show the average error for ten measurements.

Then, the fiber was placed in a rotating clamp and rotated from 0° to 50° degrees in 5°-degree steps. An intensity-based automatic image registration algorithm was used to retrieve the rotation angle of the fiber through the images acquired by the camera. Intensity-based automatic image registration is an iterative process that requires a specified pair of images, a metric, an optimizer, and a transformation type. The image similarity metric takes two images and returns a scalar value that describes their similarity. The optimizer defines the method of minimizing or maximizing the similarity metric. The transformation type defines the type of 2D transformation that aligns the misaligned image (known as *the moving image*) with the reference image (or *fixed image*) [43]. In this case, a rigid transformation was chosen, since it includes translation and rotation. Registering the image of the rotated fiber (moving image) and the image of the fiber in the starting position (fixed image), it was possible to obtain the rotating angles. Figure 6a shows how the images were processed using the registration algorithm; the two overlapped images are on the left, and the result of the algorithm processing is on the right. The rotation measurements were repeated 10 times: Figure 6b shows the correspondence between the rotating angles and the retrieved angles, using the method described above, and the average error. It was possible to measure the rotation angle with an accuracy of 0.01° in our lab condition. The error in the measurement is due to the noise present in the image when captured, which affects the result of the algorithm used to retrieve the angle.

## 5. Conclusions

This paper describes the fabrication, simulation, and experimental validation of a bend direction and rotation sensor employing a three-lobe fiber. A system normally used for textile fiber fabrication was employed to fabricate the PMMA filament with a custom-made extrusion die. The extrusion process was optimized to obtain the lowest optical loss. The guided modes in the obtained plastic fiber were simulated on Lumerical MODE software. The light field shift due to bending was addressed in the simulations. This shift was experimentally monitored by interrogating the fiber in transmission. The circular asymmetry of the fiber allows retrieving the direction of bending in space and also the rotation angle by processing the images with an intensity-based image registration algorithm.

## Figures and Tables

**Table 1:** Properties of Plexiglass 6N and 8N.

Properties:	Plexiglass 6N	Plexiglass 8N
Tensile modulus (MPa)	3200	3300
Stress @ break (MPa)	67	77
Strain @ break (%)	3	5.5
Refractive index	1.49	1.49
Density (g/cm^3^)	1.19	1.19
Melt volume rate (cm^3^/10 min)	12	3

**Table 2:** Optical fiber losses (α) for plastic optical fibers (POFs) fabricated with different extrusion parameters.

Sample ID	Sample 1		Sample 2		Sample 3	
Material Grade	Plexiglass 8N		Plexiglass 8N		Plexiglass 6N	
PMMA pump velocity (rpm)	12	12	8	8	10	10
Spool speed (m/min)	18	18	20	20	24	24
α (dB/m)	27.66	28.18	25.29	19.61	21.58	20.28
Mean value (dB/m)	27.92		22.45		20.93	
